# Supplementary figures and images for: Design, development and optimization of sustained release floating, bioadhesive and swellable matrix tablet of ranitidine hydrochloride
Source: PLoS One. 2021 Jun 25;16(6):e0253391. doi: 10.1371/journal.pone.0253391 (PMC8232414; doi:10.1371/journal.pone.0253391)

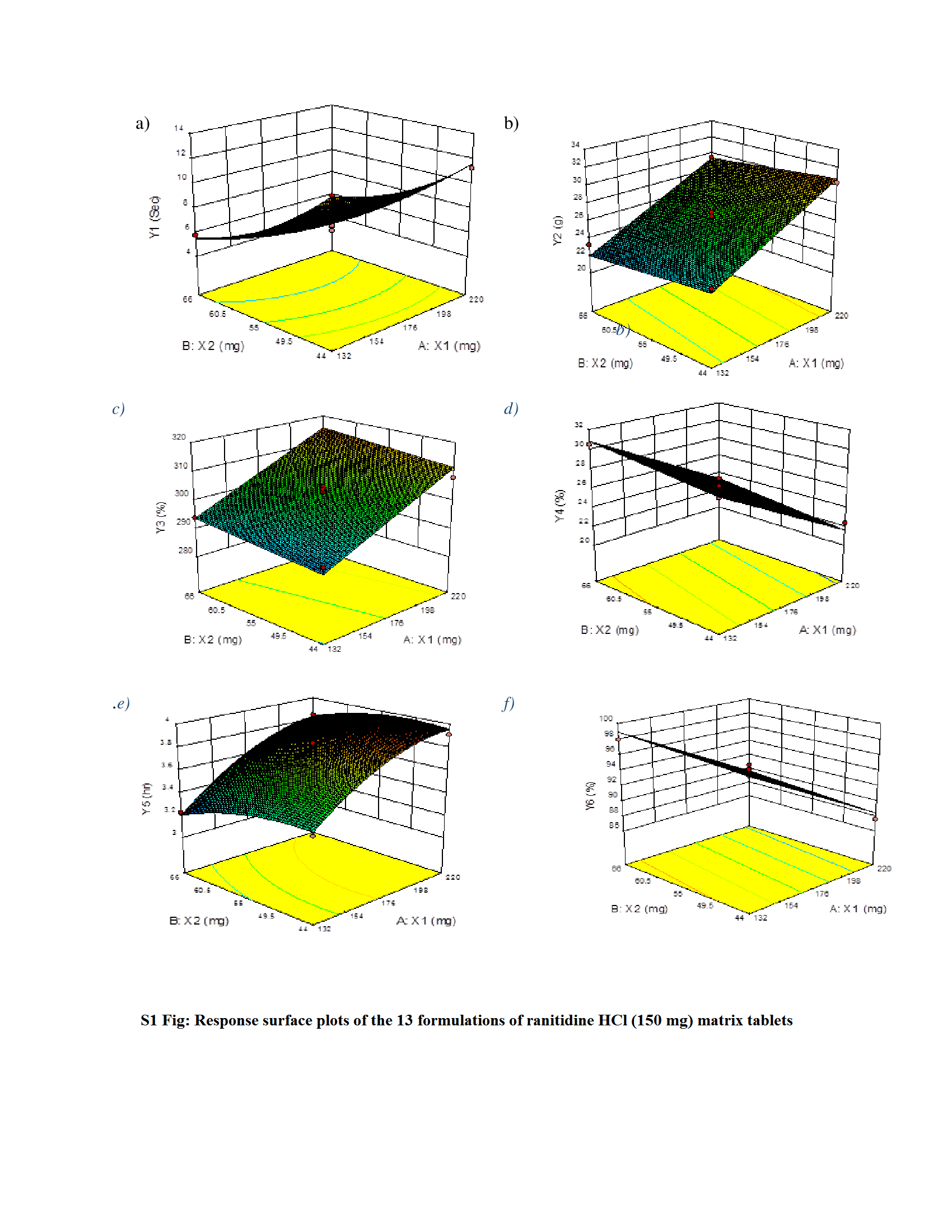

Supplement: S1 Fig — Response surface plots of the 13 formulations of ranitidine HCl matrix tablets showing the effect of concentration of HPMC/NaCMC (3:1) (X1) and Sodium Bicarbonate (X2) on a) floating lag time (Y1), b) bioadhesive strength (Y2), c) swelling index (Y3), d) cumulative drug release at 1 h (Y4), e) t50% (Y5) and f) drug release at 12 h (Y6). (TIFF) [file pone.0253391.s001.tiff]

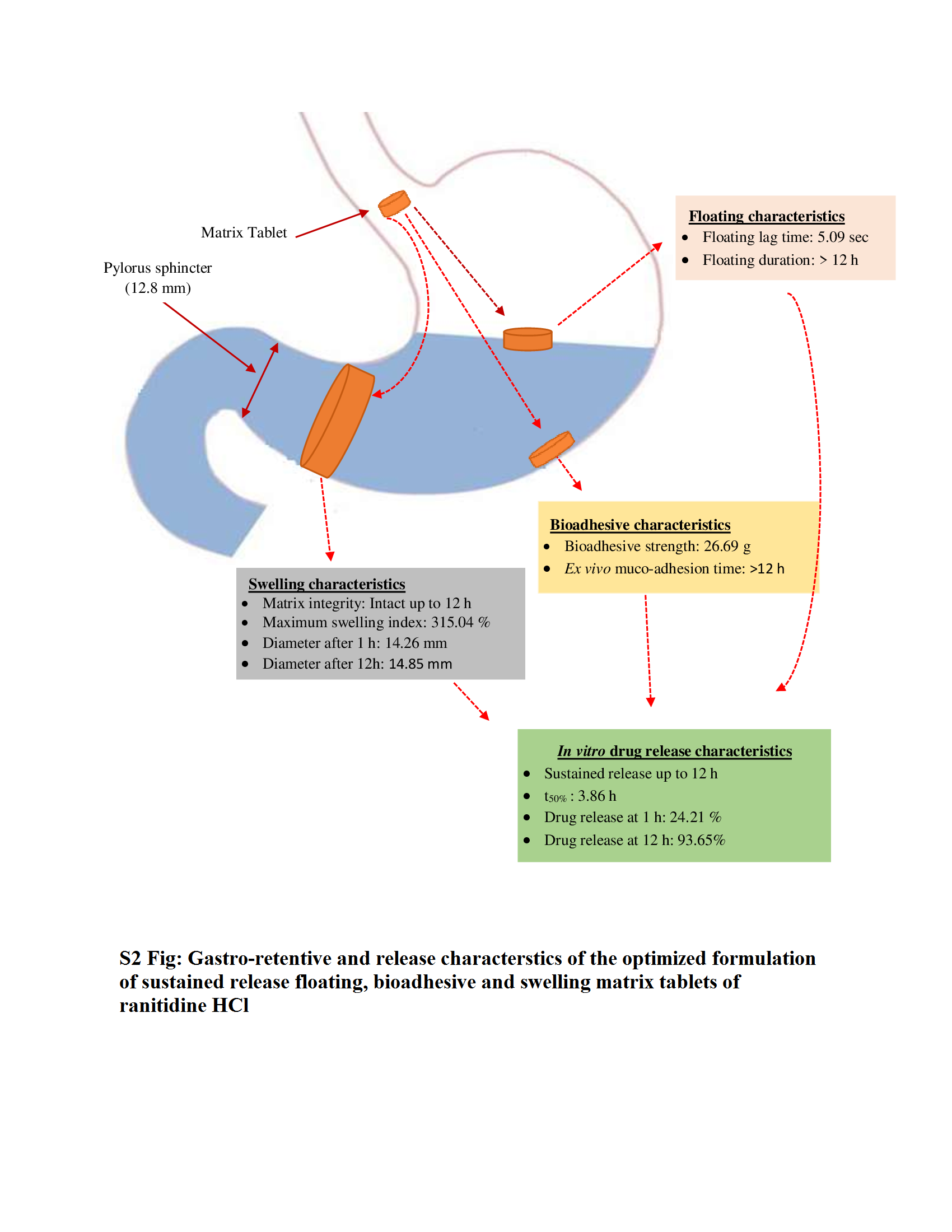

Supplement: S2 Fig — (TIFF) [file pone.0253391.s002.tiff]
