# Supplementary material for: Design, development and optimization of sustained release floating, bioadhesive and swellable matrix tablet of ranitidine hydrochloride
Source: PLoS One. 2021 Jun 25;16(6):e0253391. doi: 10.1371/journal.pone.0253391 (PMC8232414; doi:10.1371/journal.pone.0253391)
Supplement: S1 Table — (DOCX) [file pone.0253391.s003.docx]

**S1 Table.** Raw data for some *c*haracteristic properties of floating, bioadhesive and swelling sustained release matrix tablets of ranitidine HCl (150) (raw data used to create Table 3).

| Parameter | Experiment No | Formulation code/ Individual results | | | | | | | | | | | | |
| --- | --- | --- | --- | --- | --- | --- | --- | --- | --- | --- | --- | --- | --- | --- |
|  |  | F1 | F2 | F3 | F4 | F5 | F6 | F7 | F8 | F9 | F10 | F11 | F12 | F13 |
| Hardness (N) | 1 | 63 | 65 | 63 | 63 | 65 | 62 | 67 | 67 | 61 | 63 | 66 | 61 | 64 |
|  | 2 | 62 | 66 | 62 | 63 | 64 | 61 | 68 | 66 | 60 | 64 | 65 | 61 | 64 |
|  | 3 | 64 | 65 | 63 | 64 | 66 | 62 | 69 | 66 | 60 | 65 | 66 | 62 | 63 |
|  | 4 | 62 | 65 | 62 | 63 | 63 | 61 | 68 | 66 | 61 | 63 | 65 | 60 | 63 |
|  | 5 | 61 | 65 | 61 | 64 | 64 | 62 | 68 | 65 | 61 | 65 | 65 | 61 | 64 |
|  | 6 | 62 | 66 | 62 | 63 | 64 | 62 | 69 | 67 | 60 | 64 | 65 | 62 | 64 |
|  | 7 | 62 | 67 | 62 | 63 | 65 | 61 | 68 | 66 | 62 | 64 | 66 | 63 | 63 |
|  | 8 | 62 | 65 | 62 | 63 | 64 | 61 | 67 | 67 | 59 | 64 | 65 | 62 | 64 |
|  | 9 | 62 | 65 | 63 | 63 | 65 | 62 | 66 | 67 | 61 | 64 | 67 | 61 | 63 |
|  | 10 | 62 | 65 | 61 | 62 | 64 | 61 | 68 | 67 | 59 | 65 | 64 | 62 | 65 |
| Friability (%) | 1 | 0.31 | 0.42 | 0.32 | 0.43 | 0.51 | 0.46 | 0.44 | 0.42 | 0.38 | 0.56 | 0.16 | 0.21 | 0.46 |
|  | 2 | 0.32 | 0.41 | 0.33 | 0.42 | 0.51 | 0.42 | 0.41 | 0.45 | 0.24 | 0.51 | 0.32 | 0.16 | 0.42 |
|  | 3 | 0.31 | 0.41 | 0.31 | 0.42 | 0.5 | 0.38 | 0.38 | 0.38 | 0.31 | 0.46 | 0.16 | 0.15 | 0.42 |
| Thickness | 1 | 3.89 | 3.96 | 3.98 | 3.92 | 3.96 | 4.02 | 3.99 | 4.02 | 3.99 | 3.98 | 3.97 | 3.99 | 4.01 |
|  | 2 | 3.88 | 3.95 | 3.89 | 3.91 | 3.95 | 4.01 | 3.98 | 4.01 | 4.1 | 3.98 | 3.96 | 4.01 | 4.01 |
|  | 3 | 3.89 | 3.94 | 3.99 | 3.91 | 3.95 | 4.15 | 3.97 | 4.03 | 3.98 | 3.98 | 3.98 | 4.01 | 3.98 |
|  | 4 | 3.88 | 3.96 | 3.95 | 3.92 | 3.95 | 4.2 | 3.98 | 4.05 | 4.02 | 3.97 | 3.97 | 4.02 | 4.01 |
|  | 5 | 3.89 | 3.95 | 3.95 | 3.89 | 3.95 | 4.15 | 3.99 | 4.02 | 3.96 | 3.98 | 3.99 | 3.98 | 4.01 |
|  | 6 | 3.89 | 3.95 | 3.91 | 3.89 | 3.94 | 4.24 | 3.96 | 4.04 | 3.97 | 3.98 | 3.98 | 4.02 | 3.98 |
|  | 7 | 3.89 | 3.95 | 3.96 | 3.87 | 3.95 | 4.16 | 3.98 | 4.03 | 4.02 | 3.98 | 3.97 | 4.01 | 3.99 |
|  | 8 | 3.89 | 3.96 | 3.97 | 3.98 | 3.95 | 4.25 | 3.97 | 4.02 | 3.96 | 3.99 | 3.96 | 3.98 | 4.02 |
|  | 9 | 3.88 | 3.95 | 3.99 | 3.94 | 3.95 | 4.25 | 3.98 | 4.03 | 3.99 | 3.98 | 3.98 | 3.99 | 4.01 |
|  | 10 | 3.91 | 3.94 | 3.88 | 3.85 | 3.95 | 4.19 | 3.99 | 4.04 | 4.03 | 3.98 | 3.97 | 4.03 | 4.01 |
| Assay (%) | 1 | 98.27 | 101.02 | 102.51 | 100.012 | 99.58 | 98.82 | 99.98 | 101.04 | 102.01 | 98.64 | 99.81 | 100.25 | 99.27 |
|  | 2 | 98.21 | 101.03 | 102.49 | 100.011 | 99.58 | 98.99 | 100.06 | 101.04 | 101.99 | 98.67 | 99.76 | 100.24 | 99.28 |
|  | 3 | 98.28 | 101.01 | 102.5 | 100.015 | 99.51 | 98.82 | 100 | 101.04 | 102.01 | 98.72 | 99.78 | 100.27 | 99.26 |
| Floating duration (hr) | 1 | > 24 | > 24 | > 24 | > 24 | > 24 | > 24 | > 24 | > 24 | > 24 | > 24 | > 24 | > 24 | > 24 |
|  | 2 | > 24 | > 24 | > 24 | > 24 | > 24 | > 24 | > 24 | > 24 | > 24 | > 24 | > 24 | > 24 | > 24 |
|  | 3 | > 24 | > 24 | > 24 | > 24 | > 24 | > 24 | > 24 | > 24 | > 24 | > 24 | > 24 | > 24 | > 24 |
| Matrix integrity | 1 | + | + | + | + | NI | + | + | + | + | + | + | + | + |
|  | 2 | + | + | + | + | NI | + | + | + | + | + | + | + | + |
|  | 3 | + | + | + | + | NI | + | + | + | + | + | + | + | + |
| Ex mucoadhesion time (hr) | 1 | 10.55 | > 12 | 10.10 | > 12 | 6.31 | > 12 | > 12 | > 12 | > 12 | > 12 | > 12 | > 12 | > 12 |
|  | 2 | 10.51 | > 12 | 10.05 | > 12 | 6.25 | > 12 | > 12 | > 12 | > 12 | > 12 | > 12 | > 12 | > 12 |
|  | 3 | 10.45 | > 12 | 10.01 | > 12 | 6.19 | > 12 | > 12 | > 12 | > 12 | > 12 | > 12 | > 12 | > 12 |
| Diameter after 1 hr (mm) | 1 | 14.08 | 14.11 | 14.06 | 14.52 | 13.31 | 14.72 | 14.2 | 14.36 | 14.26 | 14.21 | 14.27 | 14.3 | 14.3 |
|  | 2 | 14.09 | 14.14 | 14.05 | 14.53 | 13.36 | 14.74 | 14.19 | 14.35 | 14.22 | 14.2 | 14.26 | 14.32 | 14.29 |
|  | 3 | 14.07 | 14.17 | 14.07 | 14.51 | 13.29 | 14.73 | 14.21 | 14.37 | 14.23 | 14.19 | 14.28 | 14.33 | 14.28 |
| Diameter after 12 hr (mm) | 1 | 14.57 | 14.77 | 14.63 | 14.86 | 13.94 | 15.01 | 14.61 | 14.73 | 14.9 | 14.86 | 14.89 | 14.91 | 14.75 |
|  | 2 | 14.56 | 14.78 | 14.62 | 14.87 | 13.95 | 15.02 | 14.62 | 14.74 | 14.91 | 14.85 | 14.86 | 14.87 | 14.79 |
|  | 3 | 14.55 | 14.79 | 14.64 | 14.85 | 13.96 | 15.03 | 14.61 | 14.72 | 14.92 | 14.87 | 14.79 | 14.82 | 14.81 |
