# Supplementary material for: Design, development and optimization of sustained release floating, bioadhesive and swellable matrix tablet of ranitidine hydrochloride
Source: PLoS One. 2021 Jun 25;16(6):e0253391. doi: 10.1371/journal.pone.0253391 (PMC8232414; doi:10.1371/journal.pone.0253391)
Supplement: S2 Table — (DOCX) [file pone.0253391.s004.docx]

**S2 Table.** Raw data for swelling index of 13 formulations of ranitidine HCl (150 mg) matrix tablets (data used to plot Fig 1)

| Time | Swelling index (%) | | | | | | | | | | | | |
| --- | --- | --- | --- | --- | --- | --- | --- | --- | --- | --- | --- | --- | --- |
|  | F1 | F2 | F3 | F4 | F5 | F6 | F7 | F8 | F9 | F10 | F11 | F12 | F13 |
| 0 | 0 | 0 | 0 | 0 | 0 | 0 | 0 | 0 | 0 | 0 | 0 | 0 | 0 |
| 1 | 76.65 | 82.63 | 72.35 | 75.36 | 75.36 | 90.59 | 74.25 | 76.35 | 83.65 | 74.16 | 76.21 | 76.38 | 80.46 |
| 2 | 124.96 | 116.26 | 124.98 | 118.24 | 106.35 | 134.18 | 125.35 | 115.68 | 125.35 | 114.24 | 112.25 | 116.35 | 123.32 |
| 3 | 163.27 | 158.35 | 164.38 | 162.38 | 146.46 | 174.24 | 165.24 | 159.64 | 160.11 | 158.36 | 164.25 | 160.85 | 162.34 |
| 4 | 198.25 | 186.35 | 200.86 | 195.62 | 174.25 | 206.31 | 204.21 | 187.67 | 201.24 | 182.24 | 187.24 | 185.62 | 190.35 |
| 5 | 220.31 | 210.65 | 221.05 | 221.35 | 200.31 | 234.19 | 224.54 | 209.59 | 223.26 | 208.35 | 212.24 | 209.76 | 221.09 |
| 6 | 247.96 | 234.35 | 246.82 | 240.25 | 220.41 | 254.37 | 246.24 | 234.87 | 251.02 | 230.25 | 226.21 | 237.36 | 242.35 |
| 7 | 264.32 | 268.35 | 263.65 | 272.62 | 245.32 | 279.46 | 260.35 | 264.85 | 263.17 | 262.37 | 254.24 | 267.25 | 261.34 |
| 8 | 272.35 | 289.35 | 274.65 | 295.36 | 252.32 | 300.41 | 273.35 | 286.97 | 276.81 | 281.47 | 269.29 | 284.64 | 273.65 |
| 9 | 278.35 | 295.36 | 274.89 | 304.68 | 265.62 | 310.67 | 281.24 | 294.38 | 282.96 | 289.87 | 279.35 | 292.34 | 276.37 |
| 10 | 286.63 | 305.36 | 284.65 | 310.25 | 270.06 | 315.65 | 287.26 | 304.5 | 290.32 | 300.47 | 286.38 | 304.87 | 288.37 |
| 11 | 301.25 | 308.96 | 295.36 | 315.64 | 275.02 | 320.24 | 306.67 | 301.98 | 304.324 | 304.56 | 296.24 | 305.68 | 304.38 |
| 12 | 300.26 | 310.38 | 296.84 | 317.26 | 280.04 | 325.64 | 304.96 | 307.26 | 308.64 | 305.36 | 299.85 | 312.4 | 305.26 |
