# Supplementary material for: Design, development and optimization of sustained release floating, bioadhesive and swellable matrix tablet of ranitidine hydrochloride
Source: PLoS One. 2021 Jun 25;16(6):e0253391. doi: 10.1371/journal.pone.0253391 (PMC8232414; doi:10.1371/journal.pone.0253391)
Supplement: S3 Table — (DOCX) [file pone.0253391.s005.docx]

**S3 Table.** Raw data for formulations of ranitidine HCl (150 mg) matrix tablets with the levels of independent variables and observed values for the response variables (raw data used to generate Table 4).

| Formulation code | Independent variables | | Experiment No | Observed response | | | | | | |
| --- | --- | --- | --- | --- | --- | --- | --- | --- | --- | --- |
|  | X_1_ (mg) | X_2_ (mg) |  | Y_1_(Sec) | Y_2_(g) | Y_3_ (%) | Y_4_ (%) | Y_5_ (%.h^-1/2)^ | Y_6_ (%) |  |
| F1 | 132 | 44 | 1 | 12.27 | 23.88 | 191.36 | 30.08 | 3.21 | 98.62 |  |
|  |  |  | 2 | 12.21 | 23.76 | 193.45 | 30.2 | 2.85 | 99.48 |  |
|  |  |  | 3 | 12.28 | 23.43 | 191.95 | 29.96 | 4.03 | 97.33 |  |
|  |  |  | Average | 12.25 | 23.69 | 192.25 | 30.08 | 3.36 | 98.47 |  |
|  |  |  | SD | 0.037 | 0.23 | 1.07 | 0.12 | 0.60 | 1.08 |  |
| F2 | 220 | 44 | 1 | 11.4 | 30.99 | 308.25 | 22.19 | 4.13 | 88.02 |  |
|  |  |  | 2 | 11.29 | 30.37 | 307.75 | 22.45 | 3.66 | 87.68 |  |
|  |  |  | 3 | 11.15 | 29.68 | 308 | 22.66 | 3.95 | 86.89 |  |
|  |  |  | Average | 11.28 | 30.35 | 308 | 22.43 | 3.91 | 87.53 |  |
|  |  |  | SD | 0.12 | 0.65 | 0.25 | 0.23 | 0.23 | 0.57 |  |
| F3 | 132 | 66 | 1 | 6.51 | 23.56 | 294.85 | 30.87 | 3.98 | 98.55 |  |
|  |  |  | 2 | 6.2 | 23.35 | 294.65 | 30.54 | 3.24 | 97.05 |  |
|  |  |  | 3 | 5.87 | 22.69 | 293.63 | 30.38 | 2.86 | 98.21 |  |
|  |  |  | Average | 6.19 | 23.2 | 294.37 | 30.59 | 3.36 | 97.93 |  |
|  |  |  | SD | 0.32 | 0.45 | 0.65 | 0.25 | 0.56 | 0.78 |  |
| F4 | 220 | 66 | 1 | 5.46 | 29.58 | 314.13 | 22.32 | 4.06 | 88.68 |  |
|  |  |  | 2 | 5.16 | 29.18 | 313.98 | 23.01 | 4.28 | 89.32 |  |
|  |  |  | 3 | 4.58 | 29.65 | 313.86 | 23.89 | 3.1 | 89.26 |  |
|  |  |  | Average | 5.07 | 29.47 | 313.99 | 23.07 | 3.81 | 89.08 |  |
|  |  |  | SD | 0.45 | 0.25 | 0.13 | 0.79 | 0.62 | 0.35 |  |
| F5 | 113 | 55 | 1 | 8.28 | 19.61 | 284.36 | 31.22 | 3.48 | 100.82 |  |
|  |  |  | 2 | 8.52 | 20.92 | 284.35 | 31.92 | 3.18 | 99.86 |  |
|  |  |  | 3 | 8.69 | 20.15 | 283.42 | 31.48 | 2.78 | 99.58 |  |
|  |  |  | Average | 8.50 | 20.23 | 284.04 | 31.54 | 3.14 | 100.08 |  |
|  |  |  | SD | 0.21 | 0.67 | 0.54 | 0.35 | 0.35 | 0.65 |  |
| F6 | 238 | 55 | 1 | 6.2 | 32.58 | 320.54 | 21.84 | 4.28 | 88.14 |  |
|  |  |  | 2 | 6.45 | 32.92 | 320.16 | 21.05 | 3.7 | 87.85 |  |
|  |  |  | 3 | 6.68 | 31.98 | 319.84 | 20.56 | 3.98 | 87.58 |  |
|  |  |  | Average | 6.44 | 32.49 | 320.18 | 21.15 | 3.98 | 87.85 |  |
|  |  |  | SD | 0.24 | 0.48 | 0.35 | 0.64 | 0.29 | 0.28 |  |
| F7 | 176 | 44 | 1 | 14.12 | 27.25 | 300.63 | 24.62 | 3.91 | 92.8 |  |
|  |  |  | 2 | 13.52 | 27.56 | 300.05 | 24.12 | 4.06 | 92.31 |  |
|  |  |  | 3 | 12.98 | 26.92 | 299.34 | 23.58 | 3.52 | 91.86 |  |
|  |  |  | Average | 13.54 | 27.24 | 300.00 | 24.16 | 3.83 | 92.32 |  |
|  |  |  | SD | 0.57 | 0.32 | 0.65 | 0.52 | 0.23 | 0.47 |  |
| F8 | 176 | 66 | 1 | 4.79 | 24.41 | 306.71 | 28.58 | 3.89 | 95.61 |  |
|  |  |  | 2 | 4.12 | 24.85 | 306.02 | 28.32 | 3.11 | 95.29 |  |
|  |  |  | 3 | 3.43 | 23.95 | 305.28 | 28.02 | 3.51 | 95.02 |  |
|  |  |  | Average | 4.11 | 24.40 | 306.00 | 28.30 | 3.50 | 95.30 |  |
|  |  |  | SD | 0.68 | 0.45 | 0.71 | 0.28 | 0.39 | 0.29 |  |
| F9 | 176 | 55 | 1 | 6.72 | 27.1 | 305.83 | 26.86 | 4.44 | 94.42 |  |
|  |  |  | 2 | 6.21 | 25.8 | 304.18 | 26.32 | 3.86 | 95.12 |  |
|  |  |  | 3 | 5.68 | 26.4 | 305.11 | 25.88 | 3.24 | 94.51 |  |
|  |  |  | Average | 6.20 | 26.43 | 305.04 | 26.35 | 3.84 | 94.68 |  |
|  |  |  | SD | 0.52 | 0.65 | 0.82 | 0.49 | 0.60 | 0.38 |  |
| F10 | 176 | 55 | 1 | 6.85 | 27.65 | 302.03 | 25.78 | 3.76 | 93.86 |  |
|  |  |  | 2 | 6.51 | 26.59 | 302.26 | 24.86 | 4.01 | 94.26 |  |
|  |  |  | 3 | 6.15 | 25.48 | 301.75 | 24.46 | 3.47 | 94.78 |  |
|  |  |  | Average | 6.50 | 26.57 | 302.01 | 25.03 | 3.74 | 94.3 |  |
|  |  |  | SD | 0.35 | 1.08 | 0.25 | 0.67 | 0.27 | 0.46 |  |
| F11 | 176 | 55 | 1 | 7.42 | 27.98 | 303.18 | 25.89 | 4.21 | 94.12 |  |
|  |  |  | 2 | 7.01 | 27.08 | 303.24 | 25.49 | 3.56 | 93.68 |  |
|  |  |  | 3 | 6.59 | 26.24 | 302.75 | 25.11 | 3.74 | 93.14 |  |
|  |  |  | Average | 7.01 | 27.1 | 303.05 | 25.49 | 3.83 | 93.64 |  |
|  |  |  | SD | 0.41 | 0.87 | 0.26727 | 0.39 | 0.33 | 0.49 |  |
| F12 | 176 | 55 | 1 | 7.94 | 25.51 | 303.12 | 26.48 | 3.95 | 93.51 |  |
|  |  |  | 2 | 7.76 | 25.98 | 302.64 | 26.23 | 3.55 | 94.35 |  |
|  |  |  | 3 | 6.74 | 25.01 | 303.64 | 25.92 | 3.75 | 92.64 |  |
|  |  |  | Average | 7.48 | 25.5 | 303.13 | 26.21 | 3.75 | 93.5 |  |
|  |  |  | SD | 0.65 | 0.48 | 0.50 | 0.28 | 0.2 | 0.85 |  |
| F13 | 176 | 55 | 1 | 6.95 | 26.72 | 304.77 | 27.56 | 3.8 | 94.05 |  |
|  |  |  | 2 | 6.61 | 27.57 | 303.42 | 27.03 | 3.91 | 93.46 |  |
|  |  |  | 3 | 6.25 | 25.44 | 304.21 | 26.52 | 3.67 | 92.98 |  |
|  |  |  | Average | 6.60 | 26.58 | 304.13 | 27.03 | 3.79 | 93.49 |  |
|  |  |  | SD | 0.35 | 1.07 | 0.68 | 0.52 | 0.12 | 0.53 |  |
