# Supplementary material for: Design, development and optimization of sustained release floating, bioadhesive and swellable matrix tablet of ranitidine hydrochloride
Source: PLoS One. 2021 Jun 25;16(6):e0253391. doi: 10.1371/journal.pone.0253391 (PMC8232414; doi:10.1371/journal.pone.0253391)
Supplement: S4 Table — (DOCX) [file pone.0253391.s006.docx]

**S4 Table.** Raw data for *in vitro* release profile of 13 formulations of ranitidine HCl (150 mg) matrix tablets (raw data used to plot Fig 2).

| Time (hr) | Average cumulative release (%) | | | | | | | | | | | | |
| --- | --- | --- | --- | --- | --- | --- | --- | --- | --- | --- | --- | --- | --- |
|  | F1 | F2 | F3 | F4 | F5 | F6 | F7 | F8 | F9 | F10 | F11 | F12 | F13 |
| 0 | 0 | 0 | 0 | 0 | 0 | 0 | 0 | 0 | 0 | 0 | 0 | 0 | 0 |
| 0.25 | 12.26 | 9.28 | 11.12 | 10.97 | 19.51 | 8.06 | 12.42 | 12.38 | 15.24 | 13.68 | 12.86 | 14.25 | 11.65 |
| 0.5 | 18.65 | 15.75 | 19.86 | 17.69 | 21.23 | 14.36 | 19.24 | 19.63 | 19.84 | 19.79 | 18.29 | 18.69 | 19.05 |
| 1 | 31.52 | 20.36 | 30.41 | 23.69 | 35.42 | 18.38 | 24.26 | 27.36 | 25.42 | 24.69 | 25.65 | 28.67 | 25.35 |
| 2 | 39.26 | 26.68 | 42.24 | 30.41 | 48.24 | 24.26 | 29.86 | 32.25 | 32.24 | 34.68 | 30.08 | 38.64 | 29.43 |
| 3 | 48.59 | 36.54 | 53.24 | 39.94 | 59.05 | 32.08 | 39.58 | 40.28 | 41.57 | 44.69 | 39.08 | 50.49 | 40.08 |
| 4 | 60.12 | 43.98 | 61.51 | 47.68 | 68.43 | 40.38 | 48.28 | 50.36 | 55.27 | 54.67 | 50.13 | 58.61 | 48.61 |
| 6 | 74.22 | 56.38 | 79.21 | 58.34 | 85.12 | 52.76 | 62.09 | 62.38 | 67.49 | 65.97 | 59.49 | 69.35 | 58.32 |
| 8 | 85.24 | 68.67 | 92.11 | 70.64 | 99.98 | 63.24 | 75.07 | 74.69 | 78.64 | 77.68 | 74.16 | 80.24 | 70.35 |
| 10 | 95.43 | 78.96 | 99.34 | 81.24 | 100.00 | 74.39 | 85.02 | 86.38 | 86.67 | 85.67 | 81.24 | 87.69 | 81.62 |
| 12 | 98.28 | 88.24 | 99.95 | 94.26 | 100.01 | 84.37 | 96.21 | 95.67 | 97.86 | 96.89 | 94.26 | 100 | 93.67 |
