# Supplementary material for: Design, development and optimization of sustained release floating, bioadhesive and swellable matrix tablet of ranitidine hydrochloride
Source: PLoS One. 2021 Jun 25;16(6):e0253391. doi: 10.1371/journal.pone.0253391 (PMC8232414; doi:10.1371/journal.pone.0253391)
Supplement: S5 Table — (DOCX) [file pone.0253391.s007.docx]

**S5 Table.** Rate constants and correlation coefficient fits of different kinetic equations for the 13 formulations of ranitidine HCl (150 mg) matrix tablets prepared as per CCD*.*

| Formulation code | Model | | | | | | | | | | |
| --- | --- | --- | --- | --- | --- | --- | --- | --- | --- | --- | --- |
|  | Zero-order Release | | First-orderRelease | | Higuchi Square Root | | Hixson-Crowell Cubic Root | | Korsmeyer-Peppas | | |
|  | K_o_ | R^2^ | K_1_ | R^2^ | K_H_ | R^2^ | K_HC_ | R^2^ | K | R^2^ | n |
| F1 | 7.58 | 0.9206 | -0.12 | 0.9474 | 28.98 | 0.9981 | 0.26 | 0.9922 | 0.38 | 0.9961 | 0.5128 |
| F2 | 6.57 | 0.9346 | -0.07 | 0.9765 | 24.92 | 0.9974 | 0.17 | 0.9828 | 0.31 | 0.9885 | 0.5495 |
| F3 | 7.97 | 0.9047 | -0.15 | 0.9516 | 30.64 | 0.9927 | 0.30 | 0.9858 | 0.37 | 0.9889 | 0.6045 |
| F4 | 6.73 | 0.9379 | -0.07 | 0.9756 | 25.50 | 0.9978 | 0.18 | 0.9850 | 0.31 | 0.9981 | 0.5432 |
| F5 | 7.92 | 0.8657 | -0.21 | 0.9155 | 30.96 | 0.9817 | 0.34 | 0.9946 | 0.41 | 0.9867 | 0.5201 |
| F6 | 7.11 | 0.9781 | -0.07 | 0.9663 | 26.09 | 0.9780 | 0.19 | 0.9883 | 0.25 | 0.9840 | 0.5586 |
| F7 | 7.23 | 0.9552 | -0.08 | 0.9711 | 27.08 | 0.9954 | 0.21 | 0.9933 | 0.33 | 0.9930 | 0.5065 |
| F8 | 7.21 | 0.9514 | -0.09 | 0.9472 | 27.05 | 0.9934 | 0.22 | 0.9863 | 0.36 | 0.9863 | 0.4760 |
| F9 | 7.25 | 0.9587 | -0.09 | 0.9473 | 27.04 | 0.9934 | 0.21 | 0.9860 | 0.34 | 0.9842 | 0.4914 |
| F10 | 7.23 | 0.9605 | -0.08 | 0.9405 | 26.91 | 0.9885 | 0.21 | 0.9821 | 0.32 | 0.9797 | 0.4914 |
| F11 | 7.14 | 0.9618 | -0.08 | 0.9441 | 26.54 | 0.9858 | 0.20 | 0.9819 | 0.32 | 0.9670 | 0.4732 |
| F12 | 7.09 | 0.9590 | -0.08 | 0.9440 | 26.38 | 0.9858 | 0.20 | 0.9811 | 0.33 | 0.9610 | 0.4722 |
| F13 | 7.18 | 0.9591 | -0.08 | 0.9485 | 26.74 | 0.988 | 0.21 | 0.9837 | 0.31 | 0.9776 | 0.5129 |
