# Supplementary material for: Design, development and optimization of sustained release floating, bioadhesive and swellable matrix tablet of ranitidine hydrochloride
Source: PLoS One. 2021 Jun 25;16(6):e0253391. doi: 10.1371/journal.pone.0253391 (PMC8232414; doi:10.1371/journal.pone.0253391)
Supplement: S6 Table — (DOCX) [file pone.0253391.s008.docx]

**S6 Table.** Fit summary statistics for response variables of the 13 formulations of ranitidine HCl (150mg) matrix tablets prepared as per CCD.

| **Response** | **Source** | **SD** | **R-Squared** | **Adjusted**  **R-Squared** | **Predicted R-Squared** | **PRESS** |  |
| --- | --- | --- | --- | --- | --- | --- | --- |
| Floating lag time (sec) | Linear | 1.10 | 0.8762 | 0.8514 | 0.7916 | 20.42 |  |
|  | 2FI | 1.16 | 0.8762 | 0.8349 | 0.6665 | 32.69 |  |
|  | Quadratic | 0.54 | 0.9793 | 0.9646 | 0.9103 | 8.79 | Suggested |
|  | Cubic | 0.59 | 0.9824 | 0.9577 | 0.5176 | 47.28 | Aliased |
| Bioadhesive strength (g) | Linear | 0.75 | 0.9530 | 0.9436 | 0.9081 | 10.94 | Suggested |
|  | 2FI | 0.79 | 0.9533 | 0.9377 | 0.8532 | 17.48 |  |
|  | Quadratic | 0.88 | 0.9546 | 0.9222 | 0.7404 | 30.91 |  |
|  | Cubic | 0.69 | 0.9803 | 0.9527 | 0.4479 | 65.73 | Aliased |
| Swelling index (%) | Linear | 2.06 | 0.9583 | 0.9499 | 0.9157 | 86.05 | Suggested |
|  | 2FI | 2.07 | 0.9622 | 0.9496 | 0.8719 | 130.72 |  |
|  | Quadratic | 2.19 | 0.9672 | 0.9437 | 0.7947 | 209.54 |  |
|  | Cubic | 1.07 | 0.9944 | 0.9866 | 0.9607 | 40.12 | Aliased |
| Release at 1h (%) | Linear | 0.77 | 0.9515 | 0.9418 | 0.9157 | 10.38 | Suggested |
|  | 2FI | 0.81 | 0.9517 | 0.9356 | 0.8675 | 16.31 |  |
|  | Quadratic | 0.89 | 0.9550 | 0.9229 | 0.7909 | 25.75 |  |
|  | Cubic | 0.72 | 0.9789 | 0.9493 | 0.8945 | 13.00 | Aliased |
| T 50% (h) | Linear | 0.12 | 0.8358 | 0.8030 | 0.7314 | 0.23 |  |
|  | 2FI | 0.12 | 0.8387 | 0.7850 | 0.6152 | 0.33 |  |
|  | Quadratic | 0.049 | 0.9806 | 0.9668 | 0.9209 | 0.068 | Suggested |
|  | Cubic | 0.045 | 0.9885 | 0.9724 | 0.9240 | 0.065 | Aliased |
| Release at 12 hr (%) | Linear | 0.77 | 0.9672 | 0.9606 | 0.9370 | 11.38 | Suggested |
|  | 2FI | 0.75 | 0.9717 | 0.9622 | 0.9076 | 16.68 |  |
|  | Quadratic | 0.81 | 0.9745 | 0.9563 | 0.8598 | 25.32 |  |
|  | Cubic | 0.64 | 0.9888 | 0.9730 | 0.7438 | 46.27 | Aliased |
