# Supplementary material for: Design, development and optimization of sustained release floating, bioadhesive and swellable matrix tablet of ranitidine hydrochloride
Source: PLoS One. 2021 Jun 25;16(6):e0253391. doi: 10.1371/journal.pone.0253391 (PMC8232414; doi:10.1371/journal.pone.0253391)
Supplement: S7 Table — (DOCX) [file pone.0253391.s009.docx]

**S7 Table.** Summary of ANOVA showing influence of independent variables on response variables.

| **Response** | **Source** | **Sum of**  **Squares** | **df** | **Mean**  **Square** | **F**  **Value** | **p-value**  **Prob> F** | **Comment** |
| --- | --- | --- | --- | --- | --- | --- | --- |
| Floating lag time (**Y_1_**) | Model | 95.99 | 5 | 19.20 | 66.32 | < 0.0001 | Significant |
|  | X_1_ | 3.18 | 1 | 3.18 | 10.97 | 0.0129 |  |
|  | X_2_ | 82.70 | 1 | 82.70 | 285.71 | < 0.0001 |  |
|  | X_1_X_2_ | 4.225E-003 | 1 | 4.225E-003 | 0.015 | 0.9072 |  |
|  | X_1_^2^ | 1.70 | 1 | 1.70 | 5.87 | 0.0458 |  |
|  | X_2_^2^ | 9.27 | 1 | 9.27 | 32.03 | 0.0008 |  |
|  | Lack of Fit | 1.01 | 3 | 0.34 | 1.34 | 0.3806 | Not significant |
| Bioadhesive strength (**Y_2_**) | Model | 113.46 | 2 | 56.73 | 101.38 | < 0.0001 | Significant |
|  | X_1_ | 109.88 | 1 | 109.88 | 196.35 | < 0.0001 |  |
|  | X_2_ | 3.58 | 1 | 3.58 | 6.40 | 0.0298 |  |
|  | Lack of Fit | 4.24 | 6 | 0.71 | 2.09 | 0.2482 | Not significant |
| Swelling index (**Y_3_**) | Model | 978.18 | 2 | 489.09 | 114.83 | < 0.0001 | Significant |
|  | X_1_ | 944.21 | 1 | 944.21 | 221.68 | < 0.0001 |  |
|  | X_2_ | 33.97 | 1 | 33.97 | 7.98 | 0.0180 |  |
|  | Lack of Fit | 37.39 | 6 | 6.23 | 4.79 | 0.0755 | Not significant |
| Release at 1hr (**Y_4_**) | Model | 117.17 | 2 | 58.58 | 98.14 | < 0.0001 | Significant |
|  | X_1_ | 110.91 | 1 | 110.91 | 185.81 | < 0.0001 |  |
|  | X_2_ | 6.25 | 1 | 6.25 | 10.48 | 0.0089 |  |
|  | Lack of Fit | 3.51 | 6 | 0.59 | 0.95 | 0.5444 | Not significant |
| t _50%_ (**Y_5_**) | Model | 0.84 | 5 | 0.17 | 70.80 | < 0.0001 | Significant |
|  | X_1_ | 0.66 | 1 | 0.66 | 275.59 | < 0.0001 |  |
|  | X_2_ | 0.062 | 1 | 0.062 | 26.16 | 0.0014 |  |
|  | X_1_X_2_ | 2.500E-003 | 1 | 2.500E-003 | 1.05 | 0.3401 |  |
|  | X_1_^2^ | 0.10 | 1 | 0.10 | 42.33 | 0.0003 |  |
|  | X_2_^2^ | 0.035 | 1 | 0.035 | 14.49 | 0.0067 |  |
|  | Lack of Fit | 7.585E-003 | 3 | 2.528E-003 | 1.11 | 0.4435 | Not significant |
| Release at 12 h (**Y_6_**) | Model | 174.69 | 2 | 87.34 | 147.30 | < 0.0001 | Significant |
|  | X_1_ | 171.51 | 1 | 171.51 | 289.24 | < 0.0001 |  |
|  | X_2_ | 3.18 | 1 | 3.18 | 5.36 | 0.0431 |  |
|  | Lack of Fit | 4.59 | 6 | 0.76 | 2.28 | 0.2219 | Not significant |

X_1_: HPMC:NaCMC (3:1)

X_2_: NaHCO_3_
