# Supplementary material for: Design, development and optimization of sustained release floating, bioadhesive and swellable matrix tablet of ranitidine hydrochloride
Source: PLoS One. 2021 Jun 25;16(6):e0253391. doi: 10.1371/journal.pone.0253391 (PMC8232414; doi:10.1371/journal.pone.0253391)
Supplement: S8 Table — (DOCX) [file pone.0253391.s010.docx]

**S8 Table.** Raw data for *in vitro* release profile of optimized formulation of ranitidine HCl (150 mg) matrix tablets (raw data used to plot Fig 4).

|  |  | Cumulative drug release (%) | | | | | | | | | |
| --- | --- | --- | --- | --- | --- | --- | --- | --- | --- | --- | --- |
|  | Time (hr) | 0.25 | 0.5 | 1 | 2 | 3 | 4 | 6 | 8 | 10 | 12 |
| Batch 1 | Trial 1 | 10.21 | 17.24 | 23.98 | 33.31 | 41.15 | 49.68 | 62.51 | 72.1 | 83.61 | 91.26 |
|  | Trial 2 | 10.27 | 17.49 | 24.09 | 33.18 | 41.08 | 50.05 | 62.04 | 72.3 | 83.24 | 91.08 |
|  | Trial 3 | 10.16 | 16.98 | 23.86 | 33.08 | 41.49 | 48.78 | 63.04 | 71.84 | 83.76 | 91.44 |
|  | Avg. | 10.21 | 17.23 | 23.98 | 33.19 | 41.24 | 49.50 | 62.53 | 72.08 | 83.54 | 91.26 |
|  | SD | 0.06 | 0.26 | 0.11 | 0.11 | 0.22 | 0.65 | 0.50 | 0.23 | 0.27 | 0.18 |
| Batch 2 | Trial 1 | 9.83 | 17.04 | 24.08 | 32.85 | 42.52 | 49.21 | 62.41 | 71.34 | 84.02 | 92.8 |
|  | Trial 2 | 9.96 | 16.98 | 23.86 | 32.53 | 42.35 | 48.98 | 62.28 | 71.48 | 84.35 | 91.8 |
|  | Trial 3 | 9.76 | 17.16 | 24.11 | 33.14 | 42.84 | 49.46 | 62.63 | 71.24 | 84.67 | 92.21 |
|  | Avg. | 9.85 | 17.06 | 24.01 | 32.84 | 42.57 | 49.22 | 62.44 | 71.35 | 84.35 | 92.27 |
|  | SD | 0.10 | 0.09 | 0.14 | 0.30 | 0.25 | 0.24 | 0.18 | 0.12 | 0.32 | 0.50 |
| Batch 3 | Trial 1 | 9.95 | 17.34 | 24.21 | 33.2 | 42.38 | 50.35 | 62.8 | 73.2 | 83.24 | 93.21 |
|  | Trial 2 | 10.24 | 17.16 | 24.29 | 33.04 | 42.64 | 50.16 | 62.64 | 73.38 | 83.18 | 93.41 |
|  | Trial 3 | 9.85 | 17.42 | 24.31 | 33.39 | 42.88 | 50.49 | 62.94 | 73.11 | 83.38 | 93.04 |
|  | Avg. | 10.01 | 17.31 | 24.27 | 33.21 | 42.63 | 50.33 | 62.79 | 73.23 | 83.27 | 93.22 |
|  | SD | 0.20 | 0.13 | 0.05 | 0.17 | 0.25 | 0.16 | 0.15 | 0.14 | 0.10 | 0.18 |
